# Structure of the phosphocysteine intermediate of the phosphatase of regenerating liver PTP4A1

**DOI:** 10.1016/j.jbc.2025.110251

**Published:** 2025-05-19

**Authors:** Luba Mahbub, Guennadi Kozlov, Caroline Knorn, Kalle Gehring

**Affiliations:** 1 Department of Biochemistry, McGill University, Montreal, Quebec, Canada; 2 Centre de Recherche en Biologie Structurale, McGill University, Montreal, Quebec, Canada

**Keywords:** PRL1, cysteine, phosphorylation, phosphatase, magnesium, X-ray crystallography

## Abstract

Phosphatases of regenerating liver (PRL or PTP4A) are protein phosphatases implicated in cell growth, magnesium homeostasis, and cancer metastasis. During catalysis, a phosphocysteine intermediate forms, which must undergo hydrolysis to regenerate the active enzyme. In addition to dephosphorylating substrates, PRLs act as pseudo-phosphatases and bind CBS-pair domain divalent metal cation transport mediators (CNNMs) to regulate magnesium transport. In this study, we investigate the role of PRL residues in phosphocysteine hydrolysis using mutagenesis, enzyme assays, and X-ray crystallography. Loss of an aspartic acid and cysteine in the catalytic site disrupts hydrolysis and stabilizes the phosphocysteine intermediate for weeks. We use this C49S/D72A double mutant to determine the crystal structure of the cysteine-phosphorylated form of PRL1 (PTP4A1). The structure confirms that phosphocysteine sterically interferes with CNNM binding, consistent with previous biochemical studies. *In vitro* enzyme assays reveal the aspartic acid mutation increases the initial rate of catalysis for all three PRL paralogs while the homologous mutation in the phosphatases, PTP1B and PTPN12, disrupts catalysis. This highlights the mechanistic differences between PRLs and classical protein tyrosine phosphatases. Our findings refine our understanding of PRL catalysis and identify novel mutations for investigating PRL function in cancer and magnesium homeostasis.

Phosphatases of regenerating liver (PRL or PTP4A) are a small family of three cysteine-based phosphatases (also known as PTPs). PRL1 was first discovered as an immediate-early gene upregulated in regenerating rat liver (1). Interest in the family grew when PRL3 was shown to be associated with metastatic colon cancer (2). All three phosphatases promote cell proliferation and migration and are overexpressed in diverse tumors in both cellular and animal models (3, 4).

PRLs employ a two-step catalytic mechanism: (i) formation of a phosphoenzyme intermediate by dephosphorylating substrate and (ii) hydrolysis of the phosphocysteine intermediate and release of free phosphate (5). The catalytic site is highly conserved and formed by a phosphate-binding loop (P-loop) and a WPD acidic loop (6, 7, 8) (Fig. 1). The P-loop contains an arginine (R110; numbering for PRL1/PRL3) that coordinates phosphate binding and a cysteine (C104) that forms a phosphocysteine intermediate. A second cysteine (C49) can form a disulfide bond with the catalytic cysteine to inhibit activity (6). This may also serve to protect the phosphatases from irreversible oxidation (9).

**Figure 1:**
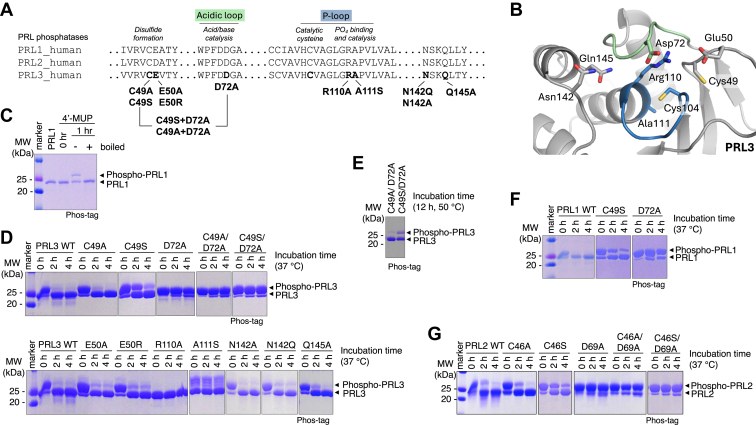
**Identification of mutants that stabilize the PRL phosphocysteine intermediate.***A*, sequence alignment of the three human PRLs and the PRL3 mutations tested. The catalytic site of PRLs consist of an acidic loop (*green*) and a phosphate-binding P-loop (*blue*). Constructs lacked the C-terminal prenylation motif. *B*, PRL3 catalytic site indicating residues selected for mutagenesis (PDB 5TSR with residue 104 shown as cysteine). *C*, Phos-tag SDS-PAGE detection of PRL1 cysteine phosphorylation following 1 h incubation with synthetic substrate, 4′-MUP. Cysteine phosphorylation is identified by boiling. *D*, phosphocysteine hydrolysis of PRL3 mutants observed over 4 h at 37 °C after removal of excess substrate. *E*, persistence of cysteine phosphorylation of the C49S/D72A PRL3 mutant at 50 °C. *F* and *G*, Phosphocysteine hydrolysis of PRL1 and PRL2 mutants at 37 °C. The smearing of the phosphorylated bands in the lane next to the molecular weight markers is an artifact due to EDTA in the protein standards. See Fig. S2−S4 for additional data. 4′-MUP, 4′-methylumbelliferyl phosphate; PRL, phosphatases of regenerating liver.

PRLs are unique phosphatases in that the second step of catalysis is very slow, which leads to persistent phosphorylation of the catalytic cysteine (6, 10). They are phosphorylated *in vivo*, and the level of cysteine phosphorylation varies according to the tissue and the availability of magnesium (11). Different substrates have been proposed, but the physiological origin of the phosphate remains unclear. Recombinant PRLs purified from bacteria are partially cysteine-phosphorylated, which suggests broad specificity (10, 12). PRLs are among the least active cysteine-based protein phosphatases (13) and evolutionarily unique in their remarkably stable phosphocysteine intermediate, enzyme kinetics, and low turnover rate (5, 6, 10, 14).

A number of studies link PRL oncogenicity to disrupted magnesium homeostasis and growth-promoting effects of intracellular Mg^2+^ (3, 4). CNNMs (CBS-pair domain divalent metal cation transport mediators) are a family of membrane proteins with a transmembrane domain that transports Mg^2+^ and a cytosolic domain that binds PRLs (15, 16). PRLs are prenylated at their C-terminus that directs their localization to cellular membranes, including the plasma membrane (17, 18, 19). A conserved aspartic acid residue in CNNMs binds to the PRL catalytic site as a phospho-substrate mimic with ∼10 nM affinity (10, 12, 20). The binding inhibits CNNM-mediated Mg^2+^ efflux and is sufficient to promote tumor metastasis (11). The PRL catalytic cysteine does not directly interact with CNNM but is crucial for the interaction. Its oxidation or phosphorylation reduces the affinity of CNNM binding more than 100-fold (10, 12).

Here, we used mutagenesis to assess the importance of PRL residues in the hydrolysis of the phosphocysteine intermediate. Unexpectedly, we find that the aspartic acid in the acidic loop is dispensable for the first step of dephosphorylating synthetic substrates and, when it is mutated to alanine, hydrolysis of the PRL phosphocysteine intermediate is greatly slowed. Taking advantage of this stabilization, we determined the crystal structure of the phosphocysteine intermediate of PRL1. The structure demonstrates that the phosphate group prevents CNNM-binding due to steric clash. Mutations that stabilize PRL cysteine phosphorylation present an opportunity to investigate the signaling and oncogenicity of this unique family of protein phosphatases.

## Results

### Identifying a phosphocysteine-stabilizing mutation

We used the sequence conservation and the atomic structure of PRL3 to identify residues that might affect the stability of the phosphocysteine intermediate (Fig. 1, *A* and *B*). In addition to residues in the acidic loop and P-loop, we mutated E50, which was reported to affect substrate turnover (21). Studies of the cysteine-based phosphatase PTP1B used a glutamine mutation (Q262A) to crystallize the phosphocysteine form (22). Based on structural overlays, the analogous position in PRL3 is N142 or possibly Q145, and we tested three mutations accordingly. Mutants were prepared by site-directed mutagenesis and their folding confirmed using ^1^H NMR spectroscopy (Fig. S1). PRL1 was phosphorylated using a synthetic substrate, 4′-methylumbelliferyl phosphate (4′-MUP), and the presence of phosphocysteine detected using phos-tag SDS-PAGE gels (Fig. 1*C*). Cysteine phosphorylation, which is heat-labile, was confirmed by boiling before electrophoresis.

Wildtype PRL3 remained partially phosphorylated after gel filtration to remove unreacted substrate but was completely dephosphorylated after 2 h at 37 °C (Fig. 1*D*). Two mutants, R110A and A111S, did not show initial phosphorylation (at 0 h). R110A was previously shown to abolish catalytic activity, while A111S accelerates phosphocysteine hydrolysis (6, 12). The PRL3 N142A and N142Q mutants had negligible effects on the rate of phosphocysteine hydrolysis while the Q145A slightly slowed hydrolysis (Fig. 1*D*).

Two mutations C49S (C-to-S) and D72A (D-to-A) markedly increased the lifetime of phosphocysteine. C49S showed some residual phosphorylation at 4 h, while D72A remained almost completely phosphorylated (Fig. 1*D*). The double mutant C49S/D72A showed extremely slow kinetics, and cysteine phosphorylation could be detected even after 12 h at 50 °C (Fig. 1*E*). Due to the high sequence identity and conserved catalytic site architecture among the PRLs (Fig. S2), we limited our studies in PRL1 and PRL2 to the mutations that showed phosphocysteine stabilization in PRL3. Analysis of the analogous C-to-S and D-to-A mutations in PRL1 and PRL2 confirmed that the mutations stabilize the phosphocysteine intermediates of all three paralogs (Figs. 1, *F* and *G*, and S3).

### PRL aspartic acid is dispensable for first step of catalysis

**Figure 2:**
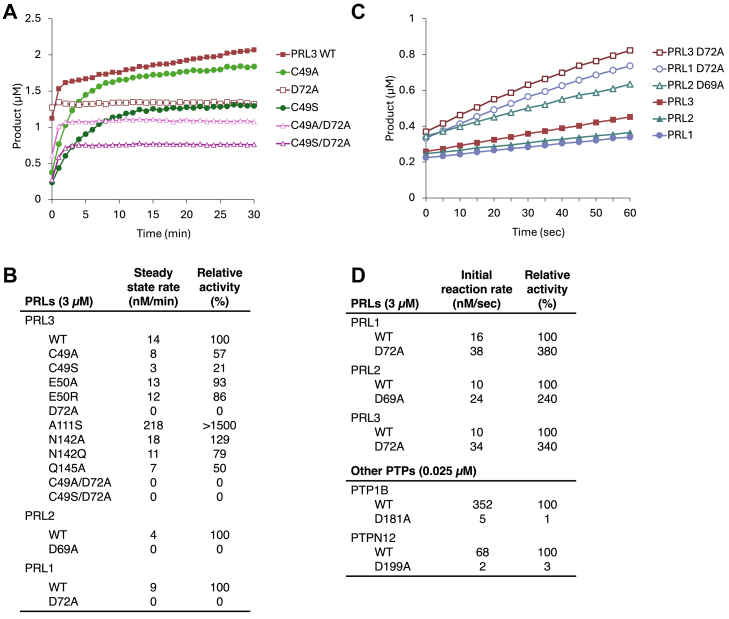
**Phosphatase activity of PRL mutants.***A*, burst kinetics of dephosphorylation of 100 μM DiFMUP by wildtype and mutant PRL3. *B*, steady-state rates measured at 20 min with 100 μM DiFMUP. *C*, initial rates of dephosphorylation of 10 μM DiFMUP by wildtype and D-to-A mutant PRLs. *D*, comparison of the initial reaction rates for PRLs [calculated from plot (*C*)] and other cysteine-based phosphatases. D-to-A mutations increase PRL activity but impair PTP1B and PTPN12 activity. Plots used to calculate the rates are shown in Fig. S5 and S7. DiFMUP, 6,8-difluoro-4-methyl-umbelliferyl phosphate; PRL, phosphatases of regenerating liver.

The mutated aspartic acid residue is in the acidic loop of the catalytic site and, in classical cysteine-based phosphatases, plays a key role in both steps of the catalytic cycle (23). However, the D-to-A mutations in PRLs did not impair activity (Fig. 1). To investigate this, we performed kinetic analysis using *in vitro* phosphatase assays of a fluorogenic substrate, 6,8-difluoro-4-methyl-umbelliferyl phosphate (DiFMUP). As previously reported, wildtype PRL3 exhibits burst-phase kinetics, which is characterized by fast initial substrate dephosphorylation followed by a slow steady-state rate (Figs. 2*A* and S5). The steady-state rate reflects the rate of phosphocysteine hydrolysis and could be seen to be close to zero for the D72A, C49A/D72A, and C49S/D72A mutants (Fig. 2*B*). The C49A and C49S mutations slowed both the initial and steady-state rates corresponding to a general loss of catalytic activity, but, surprisingly, the D72A mutation appeared to increase the initial rate (Fig. 2*A*). We repeated the enzyme assays with a lower concentration of substrate to better observe the initial kinetics (Fig. S6). E50R and N142Q showed higher initial rates but the steady-state rates were comparable to the wildtype (Figs. S6 and 2*B*). In all three PRL paralogs, the D-to-A mutation increased the initial reaction velocity 2- to 3-fold (Fig. 2, *C* and *D*). This increase could be due to a lower *K*_*M*_ for DiFMUP (possibly due to the loss of a negative charge in the active site) or a larger kinetic constant *k*_*cat*_.

For comparison, we prepared homologous mutations in two other phosphatases, PTP1B and PTPN12 (24, 25). Both enzymes are significantly more active than PRLs, which necessitated the use of 120-fold less protein in the assays. As expected, the D-to-A mutations in PTP1B and PTPN12 severely impaired dephosphorylation of DiFMUP (Figs. 2*D* and S7). We used phos-tag gels to test for the accumulation of phosphocysteine intermediates in the presence of 4′-MUP (Fig. S8). A small amount of cysteine phosphorylation was detected for PTP1B but not PTPN12. The D181A mutation did not significantly change the amount of phosphorylated PTP1B, which implies that the mutation slows roughly equally both catalytic steps: substrate dephosphorylation and hydrolysis of phosphocysteine.

### Structure of PRL1 phosphocysteine intermediate

We took advantage of the PRL mutants to capture a snapshot of the phosphocysteine intermediate by X-ray crystallography. We screened C-to-S/D-to-A double mutants as they exhibited the greatest stabilization (Fig. S9). Small crystals of PRL1 C49S/D72A were obtained with 4′-MUP in the crystallization solution to maintain cysteine phosphorylation. The best crystals diffracted to 2.6 Å resolution using synchrotron radiation (Table 1).

**Table 1:** Statistics of data collection and refinement

PDB code	9MEX
Data collection	
X-ray source	CLS CMCF-ID
Wavelength (Å)	0.953
Space group	I2_1_3
Cell dimensions	
a, b, c (Å)	148.164, 148.164, 148.164
⍺, β, ɣ (°)	90.0, 90.0, 90.0
Resolution (Å)	50–2.60 (2.64–2.60)^a^
*R*_merge_	0.104 (2.338)
*I*/σ*I*	94.6 (2.1)
Completeness (%)	100.0 (100.0)
Redundancy	20.8 (20.3)
CC_1/2_	0.962 (0.671)
Refinement	
Resolution (Å)	46.85–2.60
No. reflections	16,735
*R*_work_/*R*_free_	0.194/0.237
No. atoms	
Protein	2382
*B*-factors	
Protein	79.67
R.m.s deviations	
Bond lengths (Å)	0.005
Bond angles (°)	0.80
Ramachandran statistics (%)	
Most favored regions	97.59
Additional allowed regions	1.72
Disallowed regions	0.69

a Highest resolution shell is shown in parentheses.

**Figure 3:**
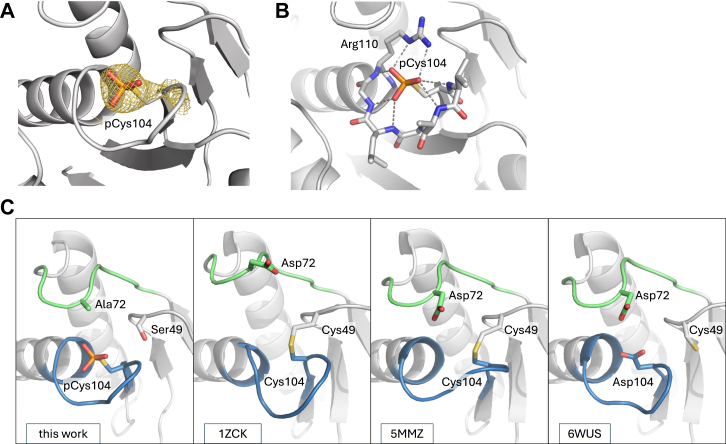
**Structure of PRL1 (7-160) C49S/D72A phosphocysteine intermediate.***A*, *2F*_*o*_*-F*_*c*_ omit map of phosphocysteine (pCys104), contoured at 2.0 σ. *B*, electrostatic interactions between the catalytic P-loop and the phosphate group. *C*, catalytic site of PRL1 in different crystal structures. Acidic loop (*green*) and P-loop (*blue*) are highlighted along with the neighboring cysteine, C49. The loops arrangement in our structure closely resembles that of PRL1 C104D (PDB 6WUS), where the orientation in oxidized PRL1 (PDB 1ZCK and 5MMZ) shows slight differences. PRL, phosphatases of regenerating liver.

The structure was solved by molecular replacement using the structure of oxidized PRL1 (7-160) (8). The asymmetric unit contains two PRL1 molecules. We did not observe the PRL1 trimers seen in earlier crystallographic studies (7, 8). PRL1 displays the characteristic cysteine-based phosphatase fold, composed of a five-stranded central β-sheet surrounded by two α-helices on one side and a four-helical bundle on the other side. The omit map clearly shows the density corresponding to the phosphate attached to the catalytic cysteine 104 (Fig. 3*A*). The positively charged side chain of the catalytic Arg110 interacts electrostatically forming a salt bridge with the negatively charged phosphate group (Fig. 3*B*). Hydrogen bonds from backbone amides in the P-loop and an alpha-helix dipole stabilize the position of the phosphate. Compared to previous PRL1 structures (8, 11, 20), the phosphocysteine active site most closely resembles that of PRL1 C104D (Fig. 3*C*).

### Phosphocysteine regulates CNNM binding

**Figure 4:**
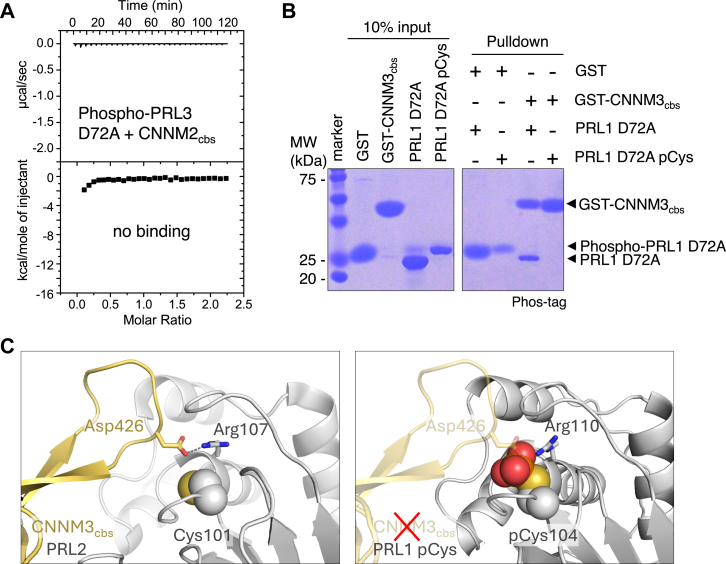
**PRL phosphocysteine prevents CNNM binding.***A*, isothermal titration calorimetry shows the cysteine-phosphorylated PRL3 D72A does not bind the CNNM2 CBS-pair domain. *B*, pulldown of recombinant PRL1 D72A by GST-fused CNNM3 CBS-pair domain shows only the nonphosphorylated form binds. *C*, structural comparison of the complex of PRL2 and CNNM3 CBS-pair domain (*left*, PDB 5K22) and phosphocysteine PRL1 (*right*). In the complex, an aspartic acid inserts into the PRL catalytic site interacting with R107. In the phosphocysteine structure, the phosphate group blocks binding. CNNM, CBS-pair domain divalent metal cation transport mediator; PRL, phosphatases of regenerating liver.

In addition to acting as phosphatases, PRLs act as pseudophosphatases binding and regulating the activity of CNNM magnesium transporters. Since a significant portion of endogenous PRLs exists in the cysteine-phosphorylated state (10, 11), their enzymatic activity is likely to have significant effect on CNNM binding. Although the catalytic cysteine does not directly interact with CNNMs, modifications of the cysteine, including phosphorylation block CNNM binding *in vitro* (10, 12). We used isothermal titration calorimetry (ITC) experiment to confirm that the cysteine-phosphorylation of PRL3 D72A prevents binding to the CBS-pair domain of CNNM2 (residues 429-584) (Fig. 4*A*). This was corroborated by GST-pulldown assays using purified recombinant proteins. The nonphosphorylated PRL3 D72A mutant was pulled down by the CNNM3 CBS-pair domain (residues 299-452), whereas the cysteine-phosphorylated form showed no binding (Fig. 4*B*). The structure of PRL2 bound to CNNM3 shows an aspartate sidechain inserts into the PRL active site interacting with the arginine in the PRL P-loop. A side-by-side comparison of the structures of the PRL–CNNM complex and the phosphocysteine intermediate shows the phosphate group precludes the CNNM aspartate entering the PRL active site (Fig. 4*C*).

## Discussion

**Figure 5:**
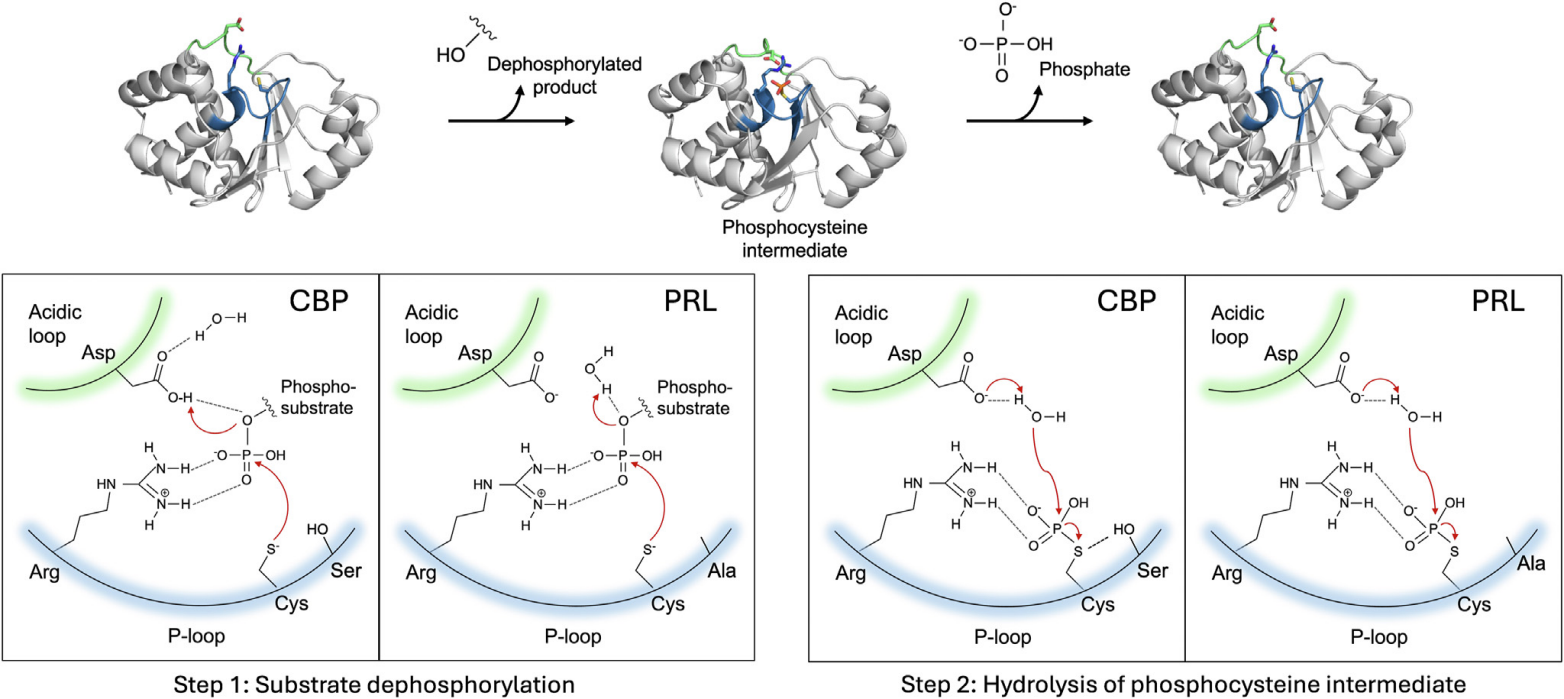
**Catalytic mechanism of PRL phosphatases.** In the first step, arginine in the P-loop coordinates phosphate binding. In other cysteine-based phosphatases, the aspartic acid acts as a general acid to promote nucleophilic attack by the catalytic cysteine to form the phosphocysteine intermediate. In PRLs, the aspartic acid is dispensable and replaced by a water molecule that activates the phospho-substrate. In the second step, the aspartic acid acts as a base to hydrolyze the intermediate. In other cysteine-based phosphatases, a serine (or threonine) residue forms a hydrogen bond with the phosphocysteine intermediate hydrolysis to make the thiolate a better leaving group (40). In PRLs, the serine is replaced by alanine. CBP, cysteine-based phosphatases; PRL, phosphatases of regenerating liver.

Cysteine-based phosphatases have a two-step catalytic mechanism involving residues from two loops (Fig. 5). In the first step, the aspartic acid in the “WPD” acidic loop acts as a general acid to prime the phosphorus atom for nucleophilic attack by a cysteine in the P-loop. Our observation that PRL aspartic acid mutants are active forces a revision of the catalytic mechanism. In PRLs, the phospho-substrate is likely activated by a water molecule in the active site. As water is a weak acid, this would account for the catalytic rate of PRLs, which is several 100-fold slower than for phosphatases such as PTP1B and PTPN12. The aspartic acid functions in the second step of catalysis where it acts as a general base to activate a water molecule to hydrolyze the phosphocysteine intermediate (26). The unusual stability of phosphocysteine in wildtype PRLs (lifetime >1 h) is due to the presence of an alanine residue in the P-loop (6). Other cysteine-based phosphatases have an active site serine or threonine that facilitates hydrolysis by making the thiolate a better leaving group or positioning the phosphocysteine for in-line attack by the nucleophilic water. Mutating the alanine to serine increases phosphocysteine hydrolysis more than 15-fold (6) (Fig. 2). In the absence of both aspartic acid and serine/threonine, the phosphocysteine intermediate of PRL1 is stable for weeks (Fig. S9).

It is notable that the PRL3 D72A mutant was previously reported to be completely inactive (6), while we observe an accelerated rate for the initial catalytic step (Fig. 2*D*). This is likely due to the rapid accumulation of the phosphocysteine form of the mutant enzyme. Wildtype PRLs are partially phosphorylated when expressed in bacteria (10). With the D72A mutation, PRL3 is almost completely phosphorylated when purified and remains so for days (Fig. S4). Without incubation to eliminate cysteine phosphorylation, the mutant appears catalytically inactive.

**Figure 6:**
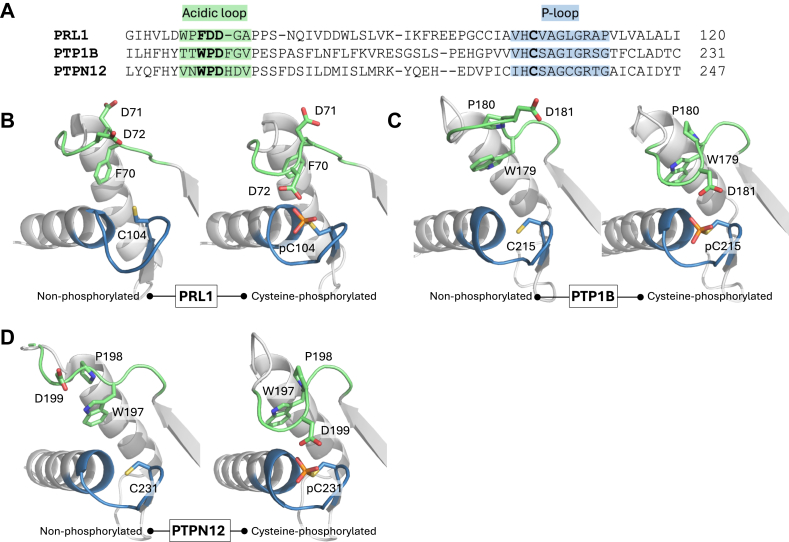
**Comparison of PRL1 with other cysteine-based phosphatases.***A*, structure-based sequence alignment of PRL1, PTP1B, and PTPN12 highlighting the catalytic acidic loop (*green*) and P-loop residues (*blue*). *B–D*, comparison of the nonphosphorylated and cysteine-phosphorylated conformations of (*B*) PRL1, PDB 1ZCK, and this work with A72 shown as aspartic acid; (*C*) PTP1B, PDB 2HNP, and PDB 1A5Y; and (*D*) PTPN12, PDB 5J8R, and PDB 5HDE. PRL, phosphatases of regenerating liver.

It is unclear why the aspartic acid in PRLs does not participate in the first step of catalysis. We compared the sequences and structures of PRL1, PTP1B, and PTPN12 in both their nonphosphorylated and cysteine-phosphorylated states (Fig. 6) (22, 27). A key difference in the enzymes lies in the sequence and orientation of the acidic loop, where the tryptophan and proline residues in PRL1 do not align with their counterparts in PTP1B and PTPN12. The acidic loop of PRLs is one residue smaller and contains two aspartic acid residues. The second aspartic acid, D71, points away from the catalytic site and does not appear to play a role in catalysis. PRL3 D71A has essentially the same the catalytic efficiency (*k*_cat_/*K*_*M*_) and rate of phosphocysteine hydrolysis as wildtype PRL3 (6).

Comparison of phosphorylated and nonphosphorylated structures show large conformational changes in the position of the acidic loop. In the nonphosphorylated structures, the loop is open with the aspartic acid far from the catalytic cysteine. The phenylalanine in the PRL1 acidic loop is positioned similarly to the tryptophan residue in PTP1B and PTPN12 and appears to anchor one side of the acidic loop. PTP1B and PTPN12 have an aromatic residue (histidine or phenylalanine) that is missing in PRLs. It is possible that the smaller loop contributes to the differences in first catalytic step. In the structures with phosphocysteine, the difference in loop size does not appear to make a difference. All three structures show the loop closed with the aspartic acid positioned above the phosphocysteine, ready to initiate hydrolysis.

The D-to-A mutation has been widely used as a substrate-trapping mutation for cysteine-based phosphatases, either alone or combined with other catalytic site mutations (28, 29). While successful in many cases, substrate-trapping experiments with PRLs have had limited success due in part to the difficulty in validating substrates given the low phosphatase activity. At saturating substrate concentrations, the level of steady-state activity is determined by the rate of phosphocysteine hydrolysis and is substrate independent (5). A second possibility is that traditional substrate-trapping mutations affect substrate affinity. PRL binding of CNNM pseudo-substrates is weakened 20-fold by D-to-A mutations and over 100-fold by catalytic cysteine to serine mutations. In the case of CNNMs, binding affinity is preserved by catalytic cysteine to aspartic acid (C-to-D) mutations. The aspartate matches the active site cysteine thiolate in size and charge but is catalytically inactive (11). Remarkably, this catalytic C-to-D mutation preserves PRL oncogenicity in a cellular model of tumor implantation (11).

In conclusion, we have identified and characterized PRL mutations that stabilize the already long-lived phosphocysteine intermediate by up to 100-fold. The mutations allowed the crystal structure of the PRL1 intermediate to be determined and refined our understanding of the catalytic mechanism of this unusual class of cysteine-based phosphatases. As the cysteine-phosphorylated forms of the proteins should persist for days *in vivo*, they may be useful tools for understanding the physiological function of PRL phosphorylation in magnesium homeostasis and tumor-associated signaling pathways that drive metastasis.

## Experimental procedures

### Expression and purification of recombinant proteins

Human phosphatase PRL1 (residues 1-169) was codon-optimized for *E. coli* expression and inserted into the pET15b vector. For crystallization, we used a previously described human PRL1 (residues 7-160) modified with a noncleavable N-terminal MGSSHHHHHH tag (11). Plasmids expressing human PRL3 (residues 1-169) and PRL2 (residues 1-163) were described previously (10, 12). Human PTP1B (residues 1-298) was subcloned as described previously (30) and was modified by inserting a His-tag fusion at the C terminus. DNA coding for phosphatase PTPN12 (residues 1-309) was chemically synthesized and cloned into vector pET15b (GenScript). Point mutants were generated with QuikChange Lighting Site-directed Mutagenesis Kit (Agilent Technologies). For insertions and double mutations, QuikChange Multi Site-directed Mutagenesis Kit (Agilent Technologies) was used. All constructs were verified by DNA sequencing. Amino acid sequences are shown in Table S1.

Cultures were grown at 37 °C in LB medium to an absorbance of 0.8, and protein expression was induced with 0.5 mM IPTG at 30 °C for 4 h. Cells were harvested and lysed in a buffer (50 mM Hepes, pH 7.6, 0.5 M NaCl, and 5% glycerol) containing 1 mM phenylmethylsulfonyl fluoride (PMSF), 0.1 mg/ml lysozyme, and 0.04% β-mercaptoethanol. His-tagged proteins were purified by affinity chromatography on Ni-NTA agarose resin (QIAGEN) and eluted with buffer containing 0.5 M imidazole. All proteins were additionally purified using Superdex-75 (GE Healthcare) size-exclusion column equilibrated with HPLC buffer containing 20 mM Hepes, pH 7.5, 100 mM NaCl, and 5 mM tris(2-carboxyethyl)phosphine hydrochloride. Expression and purification of human CNNM2 (residues 429-584) and CNNM3 (residues 299-452) were described previously (10, 31). Protein concentrations were estimated by absorbance at 280 nm using extinction coefficients of 19,940 M^−1^ cm^−1^ for PRL constructs, 46,410 M^−1^ cm^−1^ for PTP1B, 51,340 M^−1^ cm^−1^ for PTPN12, 47,330 M^−1^ cm^−1^ for GST-CNNM2 CBS, and 50,310 M^−1^ cm^−1^ for GST-CNNM3 CBS. Molecular weights were confirmed by mass spectrometry.

### Detection of cysteine phosphorylation

2 mg/ml of purified protein phosphatase in the HPLC buffer was incubated with 20 mM of 4′-MUP for 1 h at 30 °C and unreacted substrate removed by a desalting column. The stability of the phosphocysteine was characterized by SDS-PAGE with 12% Tris-glycine gels polymerized with 40 μM phos-tag reagent (Cedarlane) and 40 μM MnCl_2_. The percentage of phosphorylated proteins was quantified using densitometric analysis in ImageJ (32), calculated as ratio of phosphorylated to total protein.

### Phosphatase assay

Different degrees of cysteine phosphorylation were observed in the mutants after bacterial expression and purification. Active enzymes were regenerated by hydrolyzing the intermediate at elevated temperatures (Fig. S4). Phosphatase assays were conducted at room temperature with the fluorogenic substrate, DiFMUP, from the EnzChek Phosphatase Assay Kit (Life Technologies). Phosphatase assays (100 μl volume) were performed with 10, 25, or 100 μM DiFMUP in 20 mM Hepes pH 7.5, 100 mM NaCl, and 5 mM TCEP. Enzyme concentrations were 3 μM for PRLs and 25 nM for PTP1B and PTPN12. The change in fluorescence at 455 nm was monitored for 60 s or 30 min on a SpectraMax M5e plate reader (Molecular Devices), and the concentration of the reaction product, 6,8-difluoro-7-hydroxy-4-methylcoumarin, was calibrated with an external standard.

### Isothermal titration calorimetry

ITC experiment was performed on MicroCal VP-ITC titration calorimeter (Malvern Instruments Ltd). Cysteine-phosphorylated PRL sample was prepared by incubation with synthetic substrate 4′-MUP for 1 h at 30 °C and removal of excess of substrate *via* desalting. The syringe was typically loaded with 160 μM concentration of the ligand, while the sample cell contained 16 μM protein. The experiment was carried out at 20 °C with 29 injections of 10 μl. Results were analyzed using ORIGIN software (MicroCal) and fitted to a binding model with a single set of identical sites.

### GST-pulldown assays

The GST-pulldown protocol for recombinant proteins has been previously mentioned (33). Briefly, a slurry of 25 μl of glutathione sepharose beads (Cytiva Sweden AB) was washed twice with 1 ml of pulldown buffer (20 mM Hepes, pH 7.5, 100 mM NaCl, 5 mM tris(2-carboxyethyl)phosphine hydrochloride, and 0.02% Igepal). In between the washes, beads were sedimented by centrifuging at 13,000 rpm for 1 min at 4 °C, and supernatant was discarded. Two hundred microliter of 1 mg/ml GST-fused protein was added to the beads and incubated on ice for 15 min. The beads were washed twice with 1 ml of buffer as mentioned previously. Fifty microliter of the binding partner (1 mg/ml) was added to the beads and incubated on ice for 30 min. The proteins were eluted with 25 μl of 20 mM reduced glutathione solution after washing the beads thrice with 200 μl of pulldown buffer. 20 μl of the eluate was transferred and mixed with 5 μl of 5 × SDS loading dye (250 mM Tris pH 6.8, 10% SDS, 0.25% (w/v) bromophenol blue, 50% glycerol, and 0.14% (v/v) β-mercaptoethanol). Fifteen microliter of the sample was loaded onto the gels. Pulldown results were verified by phos-tag SDS-PAGE.

### Crystallization

Initial crystallization conditions were identified utilizing hanging drop vapor diffusion with the AmSO_4_ screen and the Classics II screen (QIAGEN). Proteolysis was minimized by including PMSF as a protease inhibitor and screening at 4 °C. The best crystals were obtained by equilibrating a 0.8 μl drop at 10 mg/ml of PRL1 (7-160) C49S/D72A, 1 mM PMSF, and 2 mM 4′-MUP in HPLC buffer mixed with 0.8 μl of reservoir solution containing 0.2 M potassium nitrate and 2.2 M AmSO_4_. Crystals grew in 7 to 10 days at 4 °C. For data collection, crystals were cryo-protected by soaking in the reservoir solution supplemented with 20% (vol/vol) glycerol.

### Structure solution and refinement

Diffraction data from single crystals of PRL1 (7-160) C49S/D72A were collected at the Canadian Light Source. Data processing and scaling were performed with HKL-2000 (34). The initial phases were determined by molecular replacement with Phaser (35), using the coordinates of the PRL1 (PDB 1ZCK) (8). The initial phases were improved by Autobuilder in PHENIX package (36). The starting protein model was then completed and adjusted with the program Coot and improved by multiple cycles of refinement, using the program phenix.refine and model refitting. At the final stage of refinement, we also applied the translation-libration-screw option (37). Refinement statistics are given in Table 1. Figures were produced using PyMOL (Schrödinger, Inc). Dali and Clustal Omega were used for structure and sequence alignments (38, 39).

## Data availability

The coordinates have been deposited with the Protein Data Bank (PDB) under the accession number 9MEX.

## Supporting information

This article contains supporting information.

## Conflict of interest

The authors declare that they have no conflicts of interest with the contents of this article.
