# Supplementary material for: Structure of the phosphocysteine intermediate of the phosphatase of regenerating liver PTP4A1
Source: J Biol Chem. 2025 May 19;301(6):110251. doi: 10.1016/j.jbc.2025.110251 (PMC12192612; doi:10.1016/j.jbc.2025.110251)
Supplement: Supporting information [file mmc1.pdf]

Supporting information for

Structure of the phosphocysteine intermediate of the phosphatase of regenerating liver PTP4A1

Luba Mahbub[1,2], Guennadi Kozlov[1,2], Caroline Knorn[1,2], Kalle Gehring[1,2]

[1]Department of Biochemistry, McGill University, Montreal QC H3G0B1, Canada

[2]Centre de Recherche en Biologie Structurale, McGill University, Montreal QC H3G0B1, Canada

<u>Table of Contents</u> **<u>Page</u>**

Table S1	Recombinant protein sequences	S-2
Figure S1	PRL3 mutants are properly folded	S-3
Figure S2	Key catalytic residues are similarly positioned across all three PRL isoforms	S-4
Figure S3	Quantification of phosphocysteine stability in PRL mutants	S-5
Figure S4	Generation of non-phosphorylated PRLs for enzyme assays	S-6
Figure S5	Phosphatase activity of PRL mutants	S-7
Figure S6	Phosphatase activity of PRL3 mutants over the first minute.	S-8
Figure S7	The acidic loop aspartate is indispensable for phosphatase activity of PTP1B and PTPN12.	S-8
Figure S8	Stable phosphocysteine intermediate is unique to PRL phosphatases.	S-9
Figure S9	Long-term stability of the PRL phosphocysteine intermediate.	S-10

Table S1. Recombinant protein sequences

Construct	Sequence (affinity tag in grey)	MW (kDa)
**PRL1 (1-169)**	MGSSHHHHHHSSGLVPRGSHMARMNRPAPVEVTYKNMRFLITHNPTNA<br>TLNKFIEELKKYGVTTIVRVCEATYDTTLVEKEGIHVLDWPFDDGAPP<br>SNQIVDDWLSLVKIKFREEPGCCIAVHCVAGLGRAPVLVALALIEGGM<br>KYEDAVQFIRQKRRGAFNSKQLLYLEKYRPKMRLRFKDSNGHRNN	21.5
**PRL1 (7-160)**	MGSSHHHHHHPAPVEVTYKNMRFLITHNPTNATLNKFIEELKKYGVTT<br>IVRVCEATYDTTLVEKEGIHVLDWPFDDGAPPSNQIVDDWLSLVKIKF<br>REEPGCCIAVHCVAGLGRAPVLVALALIEGGMKYEDAVQFIRQKRRGA<br>FNSKQLLYLEKYRPKMRLRF	18.8
**PRL2 (1-163)**	MGSSHHHHHHSSGLVPRGSHMLEMNRPAPVEISYENMRFLITHNPTNA<br>TLNKFTEELKKYGVTTLVRVCDATYDKAPVEKEGIHVLDWPFDDGAPP<br>PNQIVDDWLNLLKTKFREEPGCCVAVHCVAGLGRAPVLVALALIECGM<br>KYEDAVQFIRQKRRGAFNSKQLLYLEKYRPKMRLRFRDTNGH	21.2
**PRL3 (1-169)**	MGSSHHHHHHSSGLVPRGSHMARMNRPAPVEVSYKHMRFLITHNPTNA<br>TLSTFIEDLKKYGATTVVRVCEVTYDKTPLEKDGITVVDWPFDDGAPP<br>PGKVVEDWLSLVKAKFCEAPGSCVAVHCVAGLGRAPVLVALALIESGM<br>KYEDAIQFIRQKRRGAINSKQLTYLEKYRPKQRLRFKDPHTHKTR	21.2
**PTP1B (1-298)**	MEMEKEFEQIDKSGSWAAIYQDIRHEASDFPCRVAKLPKNKNRNRYRD<br>VSPFDHSRIKLHQEDNDYINASLIKMEEAQRSYILTQGPLPNTCGHFW<br>EMVWEQKSRGVVMLNRVMEKGSLKCAQYWPQKEEKEMIFEDTNLKLTL<br>ISEDIKSYYTVRQLELENLTTQETREILHFHYTTWPDFGVPESPASFL<br>NFLFKVRESGSLSPEHGPVVVHCSAGIGRSGTFCLADTCLLLMDKRKD<br>PSSVDIKKVLLEMRKFRMGLIQTADQLRFSYLAVIEGAKFIMGDSSVQ<br>DQWKELSHEDHHHHHH	35.5
**PTPN12 (1-309)**	MGSSHHHHHHSSGLVPRGSHMEQVEILRKFIQRVQAMKSPDHNGEDNF<br>ARDFMRLRRLSTKYRTEKIYPTATGEKEENVKKNRYKDILPFDHSRVK<br>LTLKTPSQDSDYINANFIKGVYGPKAYVATQGPLANTVIDFWRMIWEY<br>NVVIIVMACREFEMGRKKCERYWPLYGEDPITFAPFKISCEDEQARTD<br>YFIRTLLLEFQNESRRLYQFHYVNWPDHDVPSSFDSILDMISLMRKYQ<br>EHEDVPICIHCSAGCGRTGAICAIDYTWNLLKAGKIPEEFNVFNLIQE<br>MRTQRHSAVQTKEQYELVHRAIAQLFEKQLQLYEIHGAQKI	38.7
**CNNM2 (429-584)**	[GSTtag]KEELNIIQGALELRTKTVEDVMTPLRDCFMITGEAILDFN<br>TMSEIMESGYTRIPVFEGERSNIVDLLFVKDLAFVDPDDCTPLKTITK<br>FYNHPLHFVFNDTKLDAMLEEFKKGKSHLAIVQRVNNEGEGDPFYEVL<br>GIVTLEDVIEEIIKSEILDE	43.8
**CNNM3 (299-452)**	[GSTtag]DPYSDLSKGVLRCRTVEDVLTPLEDCFMLDASTVLDFGVL<br>ASIMQSGHTRIPVYEEERSNIVDMLYLKDLAFVDPEDCTPLSTITRFY<br>NHPLHFVFNDTKLDAVLEEFKRGKSHLAIVQKVNNEGEGDPFYEVLGL<br>VTLEDVIEEIIRSEILDE	43.5

[GSTtag]=MSPILGYWKIKGLVQPTRLLLEYLEEKYEEHLYERDEGDKWRNKKFELGLEFPNLPYYIDGDVKLTQSMAIIRYIAD
KHNMLGGCPKERAEISMLEGAVLDIRYGVSRIAYSKDFETLKVDFLSKLPEMLKMFEDRLCHKTYLNGDHVTHPDFMLYDALDVVL
YMDPMCLDAFPKLVCFKKRIEAIPQIDKYLKSSKYIAWPLQGWQATFGGGDHPPKSDLEVLFQGPLGS

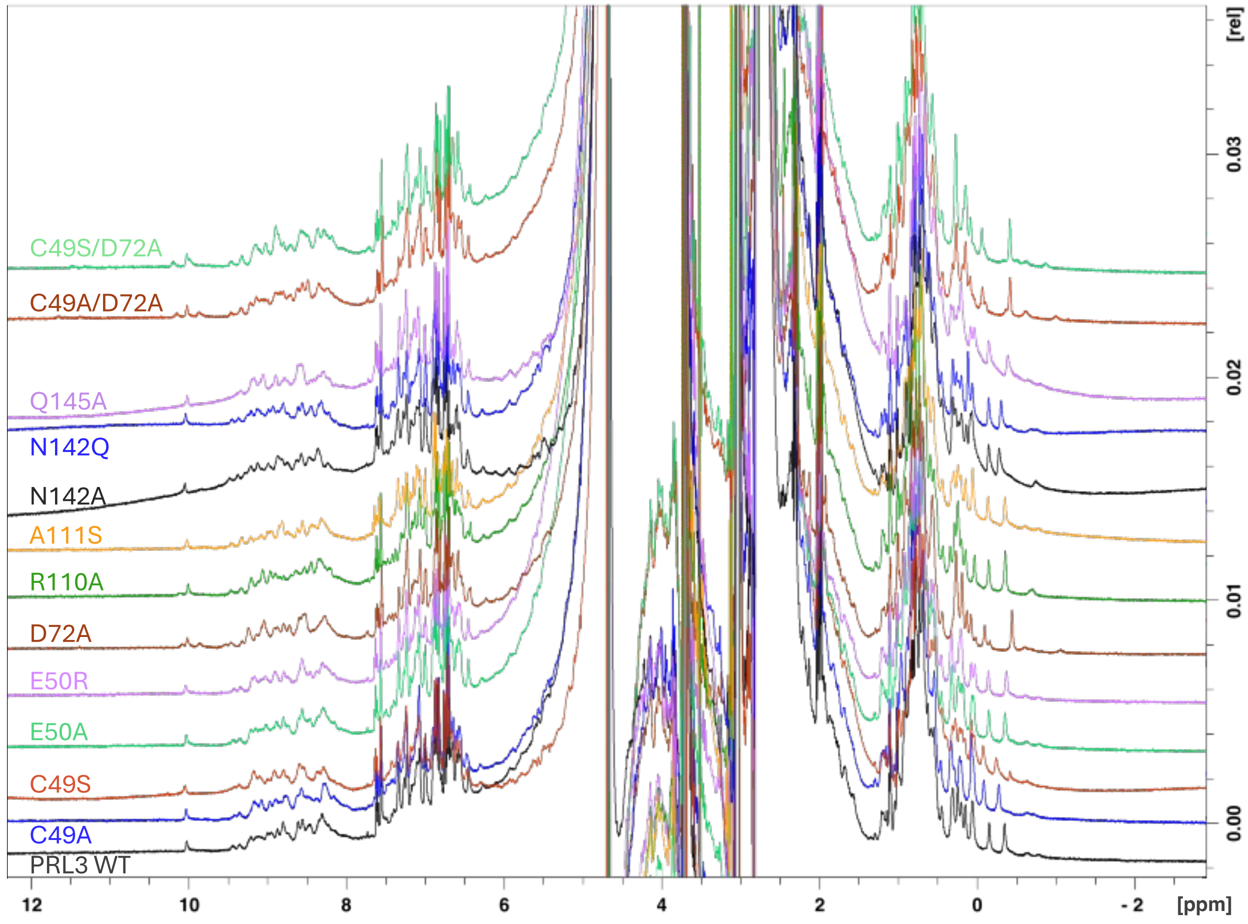

Figure S1. PRL3 mutants are properly folded. Downfield ^{1}H NMR spectra of PRL3 (residues 1-169) and mutants (C49A, C49S, E50A, E50R, D72A, R110A, A111S, N142A, N142Q, Q145A, C49A/D72A, C49S/D72A).

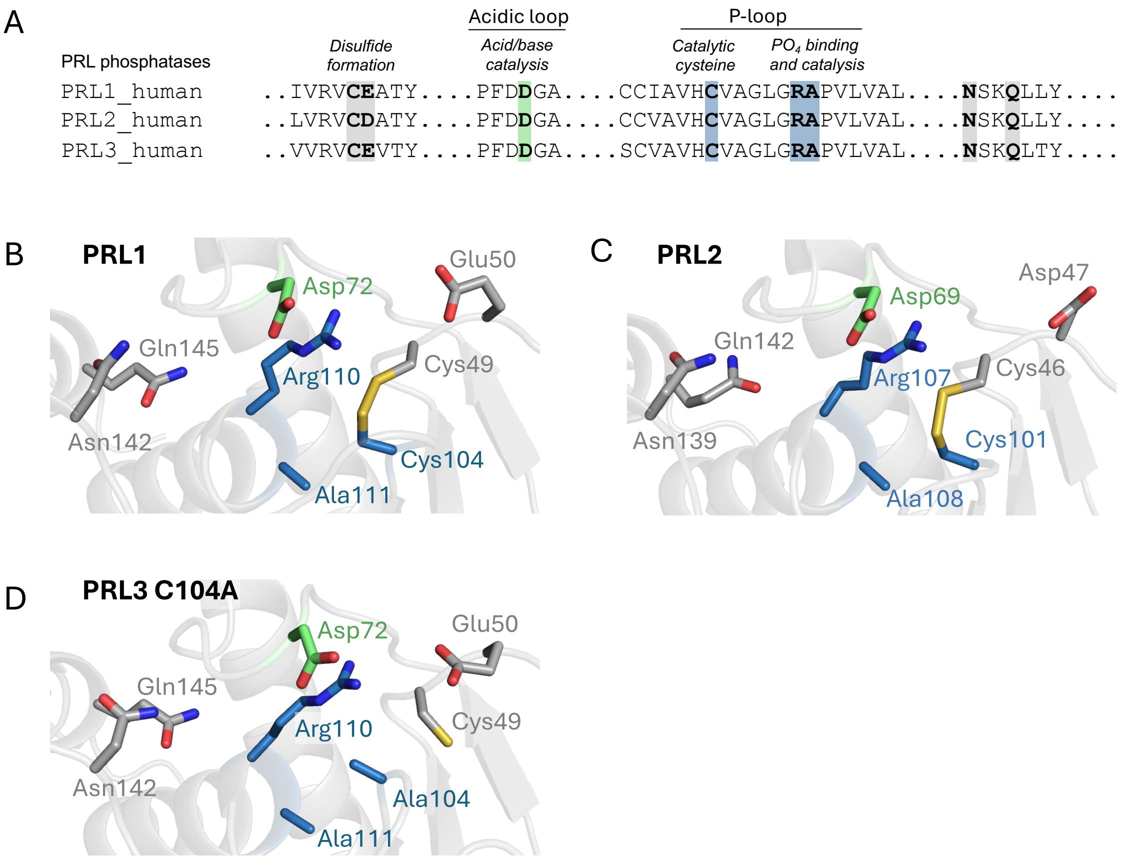

Figure S2. Key catalytic residues are similarly positioned across all three PRL isoforms. (A) Sequence alignment of human PRL1, PRL2 and PRL3, highlighting residues targeted for mutagenesis. (B-D) Structural views of the catalytic sites of (B) PRL1 (PDB 5MMZ), (C) PRL2 (PDB 5K23) and (D) PRL3 C104A mutant (PDB 5TSR) showing similar spatial arrangements of key residues. All mutations were tested in PRL3, while only selected conserved residues were analyzed in PRL1 and PRL2 due to their structural similarity.

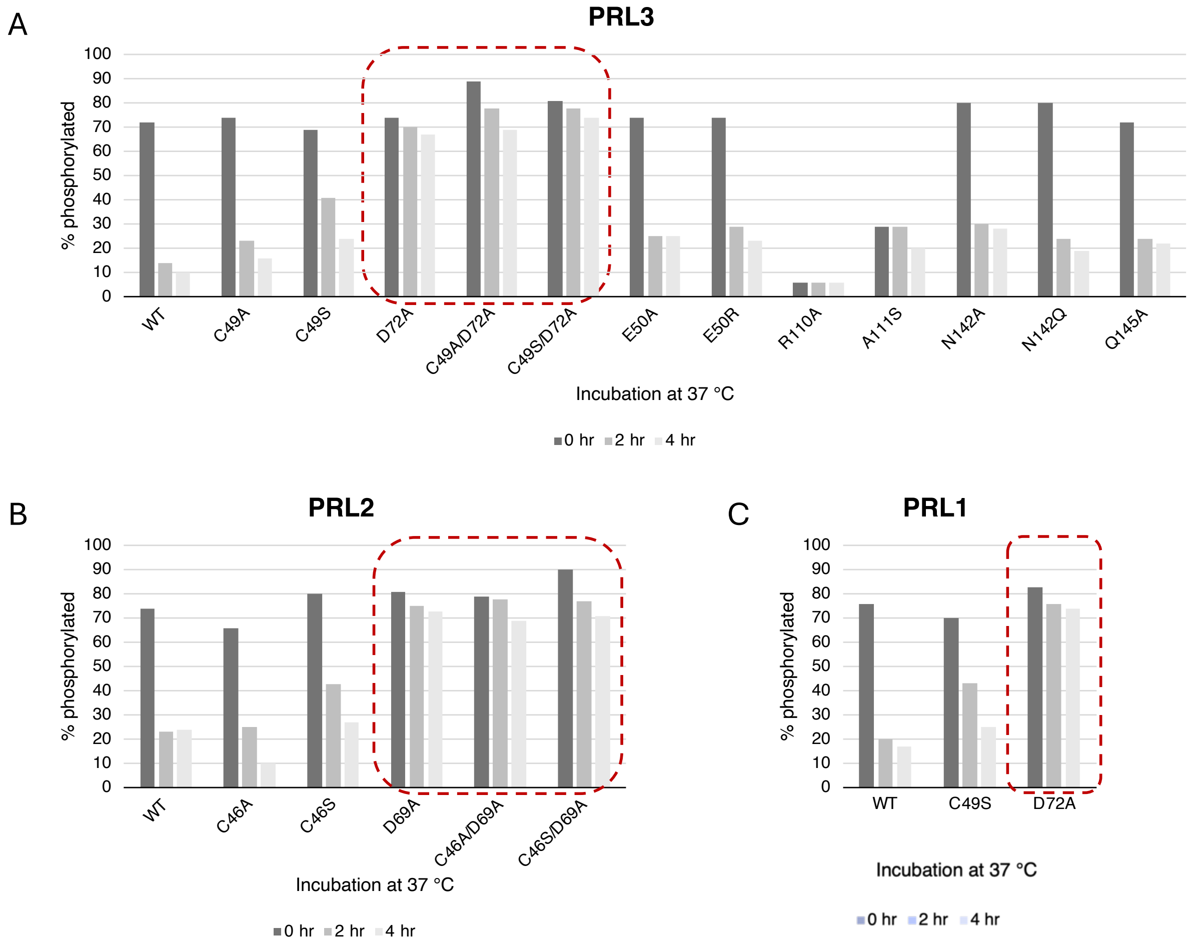

Figure S3. Quantification of phosphocysteine stability in PRL mutants. The relative levels of phosphorylated (A) PRL3, (B) PRL2, and (C) PRL1 and their mutants were quantified after incubation at 37 °C for 0, 2, and 4 hours. Band intensities were measured from the Coomassie-stained SDS-PAGE gels in Figure 1 and the percentage of phosphorylated protein from the ratio of phosphorylated to total protein. Mutants highlighted in the red dashed boxes exhibit enhanced phosphocysteine stability.

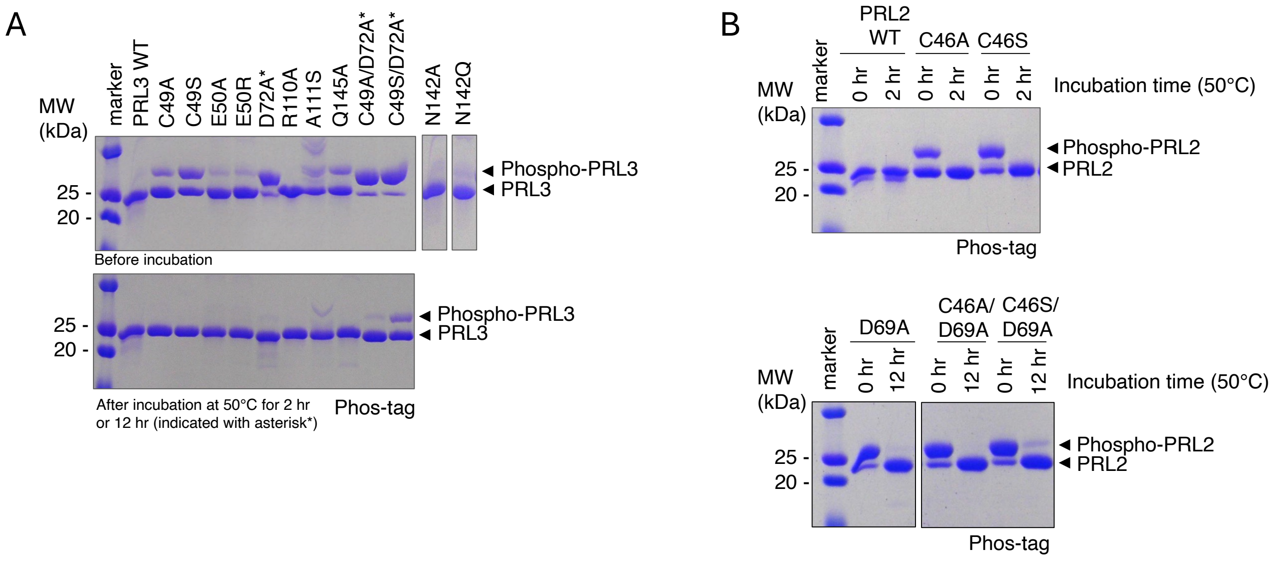

Figure S4. Generation of non-phosphorylated PRLs for enzyme assays. (A) Recombinant wild-type and mutant PRL3 purified from *E. coli* show different degrees of cysteine-phosphorylation (*top panel*). Proteins were incubated at 50 °C for 2 h or 12 h (indicated by asterisk *) to generate the non-phosphorylated forms (*bottom panel*). The N142 mutants were used without incubation as no phosphorylation was observed. (B) Cysteine-phosphorylation of recombinant wild-type and mutant PRL2 as purified from *E. coli* and after incubation to regenerate the active enzyme.

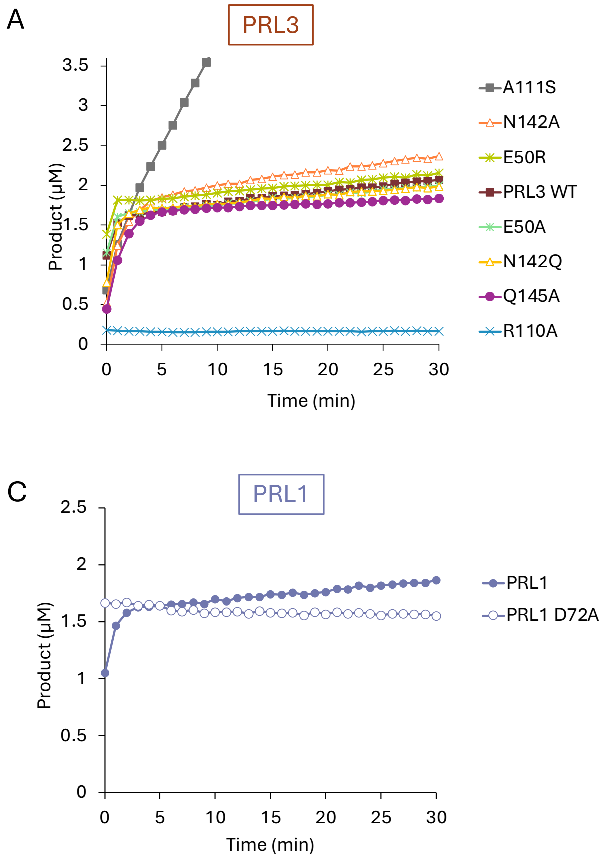

Figure S5. Phosphatase activity of PRL mutants. In presence of 100 μM DiFMUP, (A) wild-type PRL3 shows a fast kinetic step followed by a slow steady-state rate. A111S increases the steady-state rate by destabilizing the intermediate and R110A is inactive. Other PRL3 mutants show rates comparable to wild-type enzyme. (B-C) PRL2 D69A and PRL1 D72A exhibit faster initial kinetics than wild-type PRLs, reaching steady-state and maintaining a plateau, indicating no phosphocysteine hydrolysis.

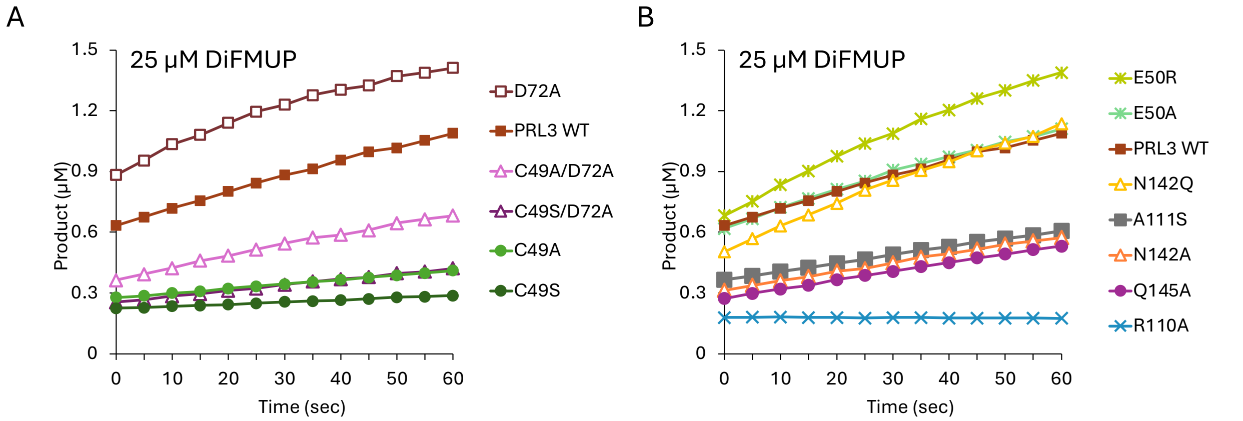

Figure S6. Phosphatase activity of PRL3 mutants over the first minute. In presence of 25 µM DiFMUP, (A) PRL3 D72A is more active than the wild-type, while the C49A and C49S mutants are less active. (B) Analysis of other mutants showed E50R and N142Q had faster activity, R110A was inactive, and the other mutants had activities comparable to the wild-type enzyme. Enzyme concentrations were 3 µM.

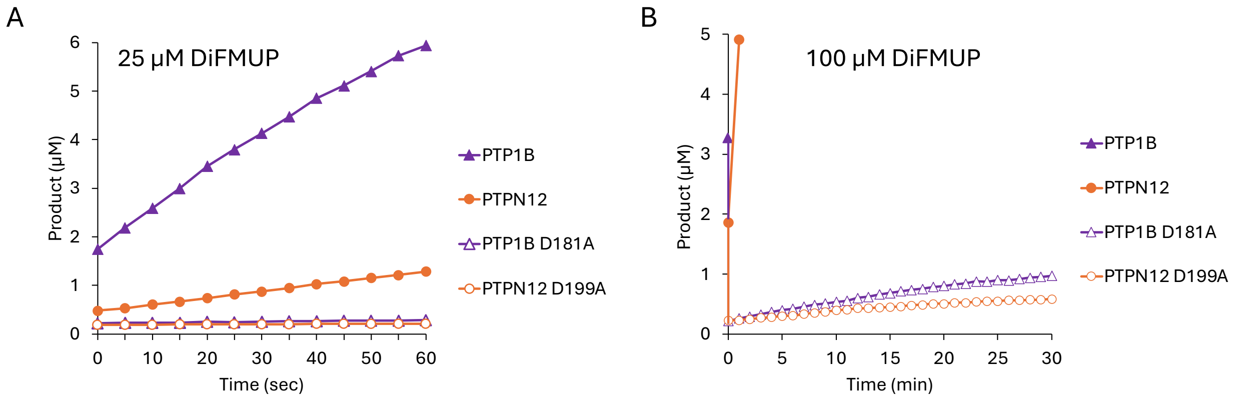

Figure S7. The acidic loop aspartate is indispensable for phosphatase activity of PTP1B and PTPN12. Substrate dephosphorylation by PTP1B or PTPN12 at (A) 25 µM and (B) 100 µM DiFMUP. The D-to-A mutations decreased the reaction rates 70-fold for PTP1B and 34-fold for PTPN12. Enzyme concentrations were 25 nM.

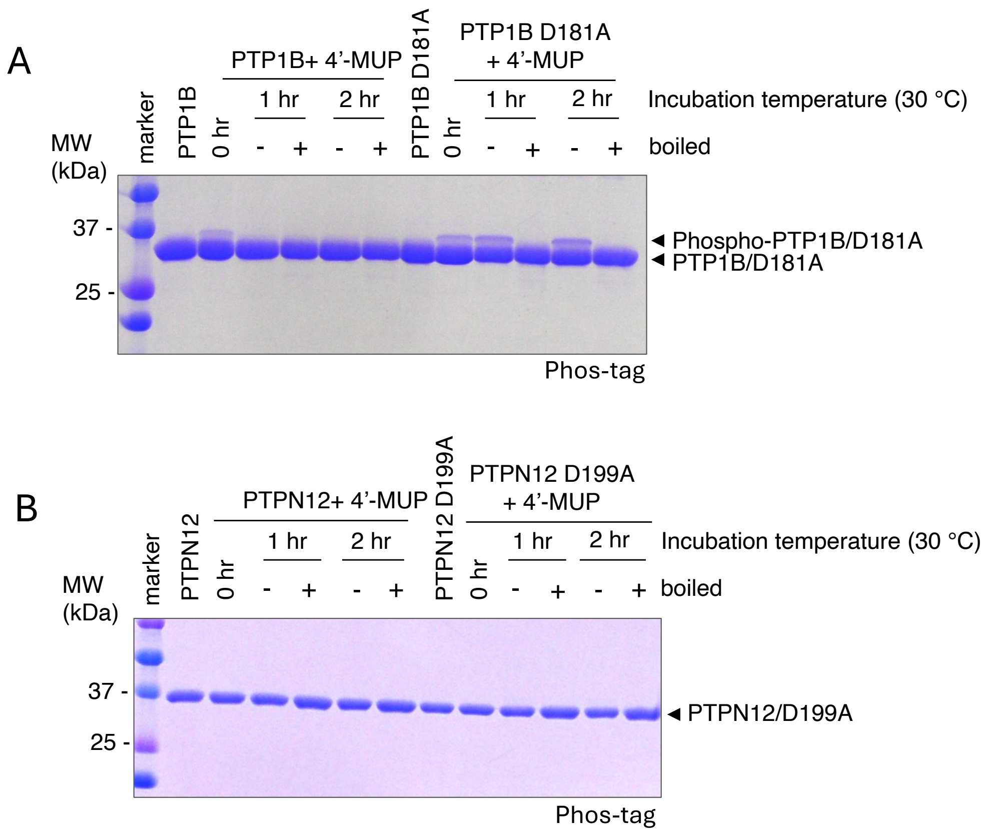

Figure S8. Stable phosphocysteine intermediate is unique to PRL phosphatases. (A) PTP1B (5 μM) forms a transient phosphocysteine intermediate in the presence of 1 mM substrate 4'-MUP. Substrate was not removed by gel filtration; rather, the disappearance of the phosphocysteine at 1 hour is likely due to enzymatic depletion of the substrate. This does not happen with the D181A mutant due its low level of catalytic activity. The fact that the PTP1B D181A mutant shows roughly the same amount of phosphocysteine as the wild-type indicates the mutation affects both steps of catalysis equally. (B) Wild-type PTPN12 and the D199A mutant (5 μM) show no accumulation of phosphocysteine under the same conditions.

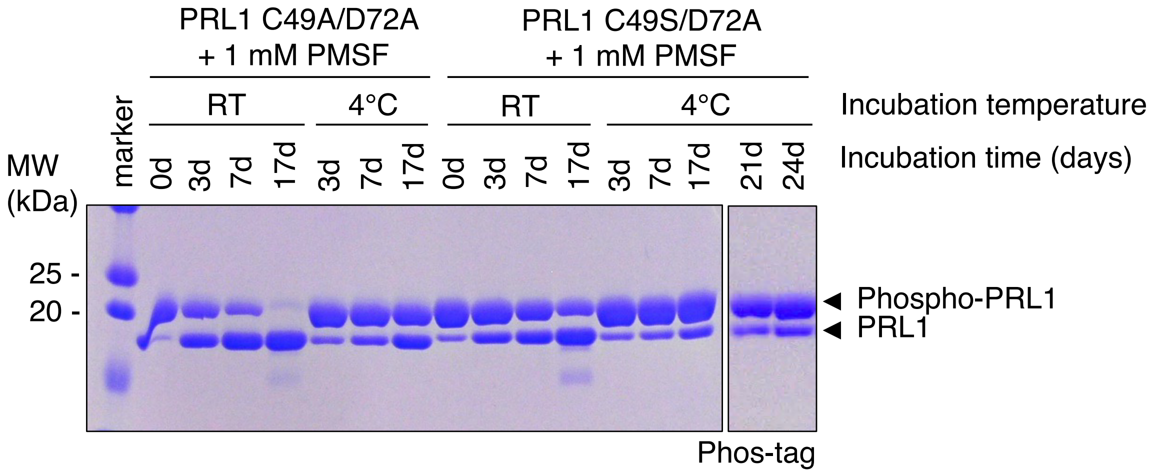

Figure S9. Long-term stability of the PRL phosphocysteine intermediate. Phospho-PRL1 (7-160) C49S/D72A is stable at 4 °C for at least up to 24 days in presence of 1 mM PMSF as protease inhibitor.
